# Cancer risk to First Nations’ people from exposure to polycyclic aromatic hydrocarbons near in-situ bitumen extraction in Cold Lake, Alberta

**DOI:** 10.1186/1476-069X-13-7

**Published:** 2014-02-12

**Authors:** Graham M Irvine, Jules M Blais, James R Doyle, Linda E Kimpe, Paul A White

**Affiliations:** 1 Department of Biology, University of Ottawa, 30 Marie Curie, Pvt, Ottawa, Ontario K1N 6 N5, Canada

**Keywords:** Risk assessment, Soil ingestion, Alberta oil sands, Cold Lake, PAH

## Abstract

**Background:**

The Alberta oil sands are an important economic resource in Canada, but there is growing concern over the environmental and health effects as a result of contaminant releases and exposures. Recent studies have shown a temporal and spatial trend of increased polycyclic aromatic hydrocarbon (PAH) concentrations in sediments and snowpack near the Athabasca oil sands operations (i.e., open pit mines), but thus far similar studies have not been done for the Cold Lake region where steam assisted gravity drainage (in situ) extraction is performed.

**Methods:**

Many PAHs are known mutagenic carcinogens, and this study measured soil and atmospheric concentrations of PAHs in the Cold Lake region to assess the excess lifetime cancer risk posed to the First Nations’ inhabitants of the region. Using both deterministic and probabilistic risk assessment methods, excess lifetime cancer risks were calculated for exposures from inhalation or inadvertent soil ingestion.

**Results:**

The mean excess cancer risk for First Nations’ people through ingestion who engage in traditional wilderness activities in the Cold Lake region was 0.02 new cases per 100,000 with an upper 95% risk level of 0.07 cases per 100,000. Exposure to PAHs via inhalation revealed a maximum excess lifetime cancer risk of less than 0.1 cases per 100,000.

**Conclusions:**

Excess lifetime risk values below 1 case per 100,000 is generally considered negligible, thus our analyses did not demonstrate any significant increases in cancer risks associated with PAH exposures for First Nations people inhabiting the Cold Lake region.

## Background

Canada has the third largest proven oil reserves in the world [1]. The oil sands in Alberta represent the vast majority of Canada’s oil reserves, and are the largest source of crude bitumen in the world. Currently, there is much speculation in the media, and within the general public, concerning the impact of the Alberta oil sands operations on the environment and human health. Some areas of the Alberta oil sands have very high background concentrations of contaminants such as polycyclic aromatic hydrocarbons (PAHs) [2-4], but there is increasing evidence that there have been significant increases in environmental levels of PAHs and metals as a result of the industrial development of the oil sands [5-11]. Additionally, there is concern in the general public and media about increased incidence of cancer and other health effects in areas impacted by the Athabasca oil sands [12,13].

There are three major oil sands regions in Alberta: (1) Athabasca (the largest); (2) Cold Lake; and (3) Peace River. The Cold Lake region, the focus of this study, is the largest *in-situ* thermal heavy oil (bitumen) extraction operation in the world, producing 54,600 barrels of bitumen each day. Currently, *in-situ* bitumen extraction accounts for 47% of oil sands extraction in Alberta, and will soon become the dominant form of oil sands extraction, overtaking open pit mining [14,15]. There is 272.3 billion barrels of known oil reserves in the Alberta oil sands, and 93% of the known reserves can be recovered with *in-situ* extraction methods. However, only 21.5 billion barrels is considered economically recoverable with *in-situ* methods [15]. *In-situ* extraction is economically viable when oil sands deposits are deeper than 100 m, and the deposits in Cold Lake are more than 400 m below the surface [14,16]. *In-situ* oil sands extraction operations in Alberta cover approximately 30 times the area that is available through surface mining, with more than twenty companies operating in the Cold Lake region [14,15]. Cold Lake is also home to the Cold Lake First Nations, which includes three reserves, as well as the City of Cold Lake [15].

Much of the published research that examined PAH levels at Alberta oil sands extraction areas has been focused on the effects of surface mining and bitumen upgrading in the Athabasca region. Consequently, the majority of this research has focused on regions with large open pit mining activities, and, in contrast, the environmental effects of *in-situ* extraction are relatively unknown. Kelly et al. [5,6] found increased heavy metals and PAH loadings in rivers downstream from bitumen upgrading facilities in the Athabasca region, and concentrations of PAHs and heavy metals were found at elevated concentrations up to 50 km away from the upgrading facilities. More specifically, Kelly et al. [6] found an estimated annual loading of ~1200 kg of particulate PAHs, with ~500 kg of dissolved PAHs within a 25 km radius from the upgrading facilities. More recently, Kurek et al. [9] noted that PAH concentrations in lake sediments in the Athabasca region have increased concomitantly with commercial bitumen extraction operations at the Athabasca oil sands. The concentrations in all lakes have increased since development of the region began, with some dated lake sediment cores showing PAH concentrations increasing tenfold relative to predevelopment. Other studies in the Athabasca region have suggested that the region has high natural background concentrations that complicate differentiation between anthropogenic influences and those that would be expected as a result of natural processes such as erosion [2-4].

Many PAHs are known mutagens or carcinogens [17] that can enter the body through a number of routes of exposure including ingestion, inhalation, and dermal contact [18]. The larger 4–6 ringed PAHs, which are typically the most carcinogenic, are highly lipophilic and readily adsorb onto particulate matter in both water and aerosols, indicating that exposure to PAHs can occur via inhalation of atmospheric particulates, ingestion of contaminated particulates (e.g., soil), or dermal contact with contaminated material (e.g., soil, sediment, water) [19-21]. Inadvertent ingestion of soil is frequently the dominant exposure pathway for soil at contaminated sites, particularly for non-volatile and semi-volatile contaminants such as carcinogenic PAHs.

This study investigated the excess lifetime cancer risk for inhabitants of the Cold Lake region associated with exposures to PAHs via inhalation of airborne particulates and the inadvertent ingestion of soil. In conjunction with our companion quantitative soil ingestion study [22], the current work, which measured PAH contamination in both soil and air, provides a unique opportunity to quantitatively evaluate the excess lifetime cancer risk to First Nations people and other residents in the Cold Lake region. More specifically, measured soil ingestion rates for inhabitants that engage in wilderness activities [22], in conjunction with environmental PAH levels, are used to estimate the excess lifetime risk of cancer for residents in the Cold Lake region. Moreover, utilizing Human Health Risk Assessments (HHRA) methods currently advocated by Health Canada [23], risk estimates for urban inhabitants of the region were also determined. We hypothesize that the excess cancer risk posed to inhabitants of the Cold Lake oil sands extraction region that are associated with potential exposures to carcinogenic PAHs in soil and air, constitutes an appreciable increase over background.

## Methods

### Study area

The study was conducted near Cold Lake, Alberta, a site approximately 300 km northeast of Edmonton, and 300 km southeast of the Athabasca oil fields. The Cold Lake region is one of the three major oil sands regions of Alberta, along with the Athabasca and Peace River deposits. The region also contains the City of Cold Lake, with a population of approximately 14,000, the Canadian Forces Base Cold Lake, and four native reserves of the Cold Lake First Nations. The aboriginal people of the Cold Lake First Nations belong to the Dene Suline tribe, whose traditional lands ranged from south of Bonnyville to the northernmost point at Peter Pond Lake, Saskatchewan.

### Soil sampling and analysis

**Figure 1:**
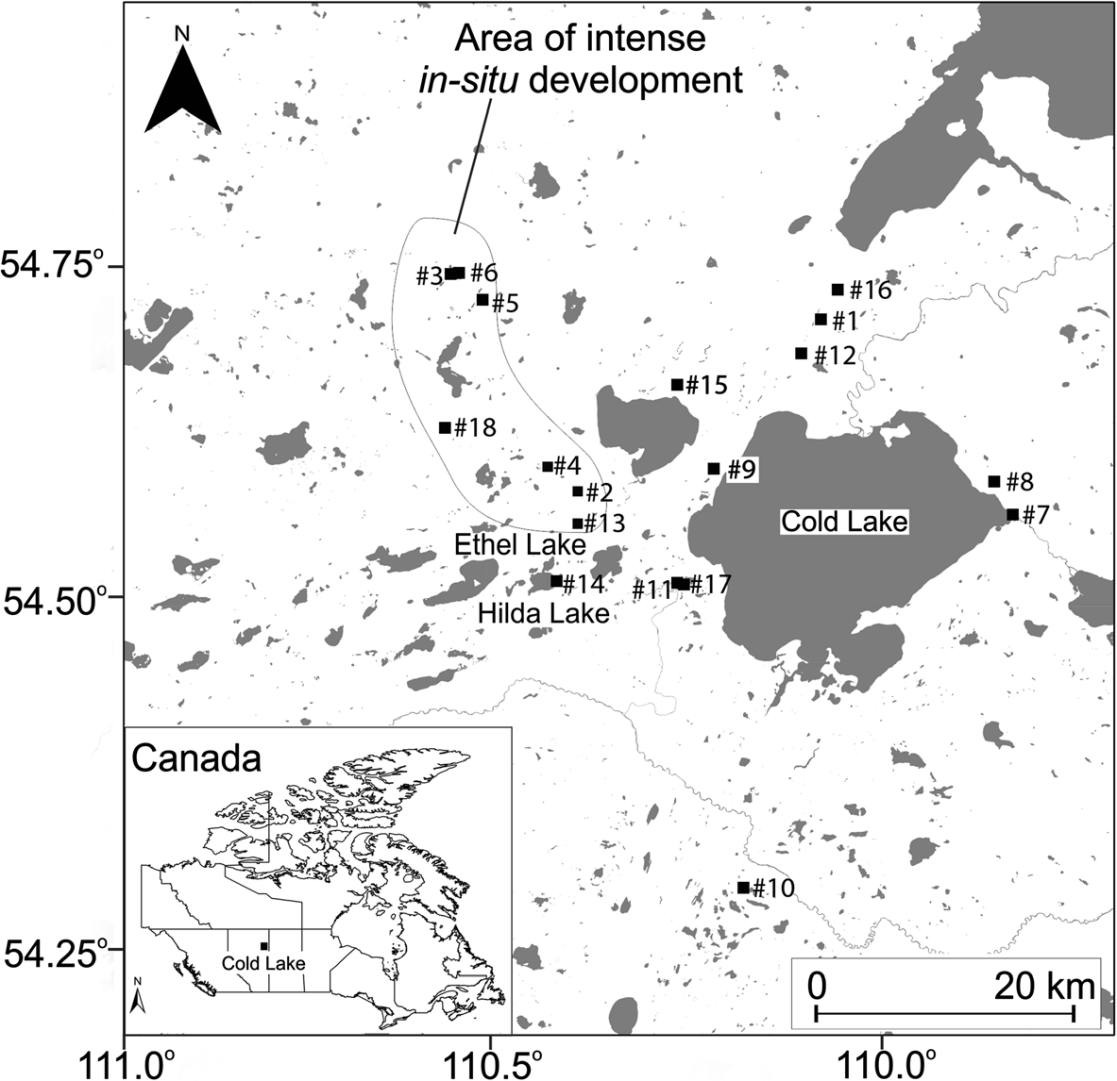
**A map of the soil sampling sites in the Cold Lake region.** Soil sampling sites are marked with a black square and numbered. Refer to Table 1.

This study collected 18 soil samples in August 2011 at 18 locations in the Cold Lake region (Figure 1). Sampling involved collection of the surficial soil horizon with a pre-rinsed spatula, removal of surface vegetation, and placement into labeled WhirlPak™ bags. Soil samples were stored in a freezer at −20°C, and shipped to the laboratory in an ice packed cooler. The sampling locations are located within the areas where participants of the soil ingestion study [22] spent significant portions of time. Soils were freeze-dried, lightly deconsolidated with a mortar and pestle, and sieved for 10 minutes to obtain the 63 μm fraction using an automated sieve shaker (Soil Test Engineering Model Cl-592B, SoilTest, Evanston, IL, USA). The 63 μm size fraction was chosen since this size fraction represents the particulate matter size that adheres to hands, and is most likely to be inadvertently ingested [24].

To analyze soil samples for PAHs, 2 g of dried soil was mixed with Hydromatrix^©^ (Agilent Technologies, Mississauga, ON), and each sample was spiked with known concentrations of US EPA 16 PAH Cocktail ^13^C (Cambridge Isotope Laboratories, Tewksbury, MA). PAHs were extracted from soil using accelerated solvent extraction (ASE 200 Accelerated Solvent Extraction System, Dionex) at 100°C using 35% acetone: 65% hexane (Omnisolv grade solvents, Fisher Scientific, Ottawa, ON). Liquid-liquid extractions were used to separate polar extracts from non-polar fractions, which were collected using hexane, 2,2,4-trimethylpentane, and saturated Na_2_SO_4_. The non-polar fractions were purified to remove pigments and sulphur using USEPA method 3640A with Envirogel columns on a preparative liquid chromatograph (Waters, Milford, Massachusetts; Agilent Technologies, Mississauga, ON). Purified samples were evaporated to 1 mL, and the PAH fraction separated on an activated 60–100 mesh Davisil 635 silica column (USEPA 3630C). The PAH fraction was analyzed with a 6890 gas chromatograph and 5973 mass spectrometer by injecting 1 μL with pulsed splitless mode at 280°C on a DB-XLB 30 m × 0.18 μm × 180 μm column (Agilent Technologies, Mississauga, ON). The oven had an initial temperature of 60°C and was held for 2 minutes, and then increased at a rate of 6°C per minute up to 300°C, and held for 10 minutes. There was a constant flow of helium at a rate of 39 cm s^-1^ for a total runtime of 52 minutes. The mass spectrometer had a transfer line temperature of 280°C, a source temperature of 230°C and quadrupole temperature of 150°C. The 16 US EPA priority PAHs and ^13^C labeled PAHs were analyzed and quantified with single ion monitoring. All PAH concentrations were calculated using isotopic dilution with labeled ^13^C PAHs, method detection limits are provided in Additional file 1: Table S1. The 16 PAHs measured, and their potency equivalency factors (PEF) are provided in Additional file 2: Table S2. Potency Equivalency Factors (i.e., PEFs) were used to convert PAH levels to BaP equivalents for assessment of carcinogenic risk [25].

### Air sampling and analysis

A high volume continuous air sampler was setup on the Cold Lake First Nations reserve adjacent to the campsite used during the companion soil ingestion study [22]. The air sampler was calibrated with a 2100 magnehelic pressure gauge using a standard high-volume sampler calibration procedure (Dwyer Instruments, Michigan City, IN). Flow through the high-volume sampler in m^3^ min^-1^ was calibrated against inches of H_2_O magnehelic readings [26]. Glass fiber filters (GFF) (Whatman 110 mm 0.7 μm pore size, Fisher Scientific, Ottawa, ON) and large polyurethane foam (PUF) cartridges (Supelco, Oakville, ON) were replaced every 24 hours. An average volume of 844.71 m^3^ of air was sampled during each 24 hour period. Prior to use, all GFFs were placed in a muffle furnace for 6 hours at 500°C to remove organic carbon, and then pre-weighed and placed in sealed containers. Methanol rinsed metal tweezers were used to remove GFFs and PUF cartridges during sampling. After sampling, GFFS and PUF cartridges were kept in a freezer at −20°C, and shipped to the laboratory in ice packed coolers.

PAH analyses were conducted as described for soil samples with the following differences. GFFs were weighed again, and placed in ASE cells that were filled with Hydromatrix^©^, PAH method detection limits for GFF samples provided in Additional file 3: Table S3. PUF cartridges were cut in half, with the top and bottom halves analyzed separately to determine breakthrough, and then placed in ASE cells that were filled with Hydromatrix^©^. PAH method detection limits shown in Additional file 4: Table S4.

### Risk assessment

The risk assessment methodology advocated by both the US EPA and Health Canada was used for assessing excess lifetime cancer risk from exposure to PAHs [27,28]. Soil ingestion rates used to calculate the dose were determined through a First Nations soil ingestion study conducted over a 13 day period at Cold Lake [22]. Deterministic assessments to assess lifetime cancer risk through ingestion of soil were conducted using Eq. 1 [27], where Cs is the concentration in the soil (mg kg^-1^), IR is the soil ingestion rate (mg d^-1^), CF is the conversion factor (10^-6^), EF is the exposure frequency, from 153 d yr^-1^ to 365 d yr^-1^, ED is the exposure duration, which is 70 years to assess lifetime cancer risk, and BW is body weight (kg). Following the recommendations of the US EPA, 80 kg was used for this study [29]. AT is the averaging time of 25550 days (365 d y^-1^ × 70 y) [27,30], and CSF is the cancer slope factor, which was 7.3 (mg kg^-1^ d^-1^)^-1^ for BaP ingestion [31]. Soil ingestion rates used to calculate dose were determined in the aforementioned 13-day soil ingestion rate study conducted at Cold Lake [22].

(1) $$\mathrm{Risk}=\frac{\mathrm{Cs}\times \mathrm{IR}\times \mathrm{CF}\times \mathrm{EF}\times \mathrm{ED}}{\mathrm{BW}\times \mathrm{AT}}\times \mathrm{CSF}$$

The assessment of excess lifetime cancer risk through inhalation of PAHs was conducted using Eq. 2 (from US EPA [32]). CA is the concentration in the air (mg m^-3^), ET is the exposure time (24 hr d^-1^), EF is exposure frequency (365 d yr^-1^), ED is exposure duration (70 yr), and AT is an averaging time of 613200 hours (24 hr d^-1^ × 365 d y^-r^ × 70 y), and IUR is the inhalation unit risk, which was 0.0033 (mg m^-3^)^-1^ (from Health Canada [31]).

(2) $$\mathrm{Risk}=\frac{\mathrm{CA}\times \mathrm{ET}\times \mathrm{EF}\times \mathrm{ED}}{\mathrm{AT}}\times \mathrm{IUR}$$

Monte Carlo simulation was employed to conduct probabilistic risk assessments following the US EPA [27] method. Eq. 1 was used with the same inputs as the deterministic risk assessments, except where indicated in the results. Cs, IR, and EF were assumed to have lognormal or uniform distributions, and all Monte Carlo simulations were set for 10,000 iterations. Oracle™ Crystal Ball (11.1.2.2.0) software was used for Monte Carlo simulations.

Excess cancer risk values were compared with literature values for four other petroleum extraction regions. More specifically, PAH concentrations for the 16 priority PAHs examined in this study were obtained from the literature [33-35], converted to BaP equivalents using PEFs (Additional file 1: Table S1), and excess lifetime cancer risk assessed (Eq. 1) for maximum and mean BaP equivalent concentrations, assuming a 20 mg d^-1^ soil ingestion rate.

Health Canada defines excess lifetime cancer risk below 1E-05 as negligible, and this threshold was used to evaluate the results obtained in this study [28,31].

## Results

### Soil PAH contamination

**Figure 2:**
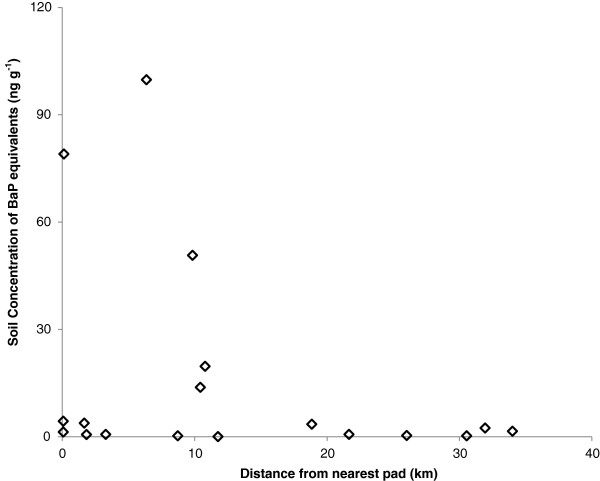
The concentration of PAHs, expressed as BaP equivalents, in soil samples collected at Cold Lake, Alberta as a function of distance from the nearest oil pad.

Eighteen soil samples were collected and analyzed to determine concentrations of 16 priority PAHs and BaP equivalents at different distances from the oil sands facilities in Cold Lake, Alberta, and results are summarized in Table 1 and Figure 2. There was no significant relationship between PAH concentration, expressed as BaP equivalents, and distance from an oil sands facility (F_(1, 16)_ = 1.73, p = 0.21). The maximum concentration of soil PAH was 99.78 ng g^-1^ of BaP equivalents sampled at 6.4 km (Figure 2, #14) from the nearest drilling pad. The second highest concentration of 79.00 ng g^-1^ was observed at the roadside adjacent to one of the oil sands facilities. The mean soil concentration of BaP equivalents was 15.70 ± 29.67 ng g^-1^, and the median concentration was 2.00 ng g^-1^. Most of the sampled soils had relatively low concentrations with 13 soil samples below 5 ng g^-1^ and 7 of these soil samples were below 1 ng g^-1^. The results obtained did not show a relationship between distance to *in-situ* oil sands facilities and soil PAH concentration.

**Table 1:** The 16 priority PAH concentrations, ΣPAH, and BaP equivalents, in soil samples collected in Cold Lake, Alberta

**Sample #**	**1**	**2**	**3**	**4**	**5**	**6**	**7**	**8**	**9**	**10**	**11**	**12**	**13**	**14**	**15**	**16**	**17**	**18**
Distance from nearest pad (km)	21.7	0.1	11.8	0.1	1.9	3.3	32	30.6	8.7	34	10.8	18.9	1.7	6.4	9.9	26	10.4	0.1
	PAH Concentration (ng g^-1^)																	
Naphthalene	-	1.9	5.1	-	27.5	-	26.4	-	-	-	100.5	-	-	-	-	-	-	-
Acenaphthylene	-	-	-	38.2	39.9	30.4	-	24.7	22.5	22.3	15.9	22.4	20.5	6.3	17.5	30.9	17.2	93.5
Acenaphthene	-	-	-	1.1	1.9	-	-	-	0.5	3.8	-	-	18.1	8.7	-	11	39.7	-
Fluorene	-	0.6	-	-	20.5	-	0.4	-	-	-	0.1	1	4.9	2.7	-	-	2.4	1.4
Phenanthrene	-	5.9	-	-	23.5	-	1.4	-	-	-	1.9	12.5	29.2	35.4	4.3	-	33.1	5.4
Anthracene	-	-	-	-	7.8	-	-	-	-	-	3.4	-	-	-	0.4	2	6.1	0.6
Fluoranthene	-	11.8	-	0.7	7.6	-	4.2	0.8	-	0.7	13.8	5.8	5.9	154.2	18.4	-	14	2
Pyrene	-	46.5	-	-	-	-	6.1	2	-	-	13.5	2.6	7	66.1	18.6	-	15.3	6.2
Benz[a]anthracene	-	4.4	-	-	-	-	0.6	-	-	0.6	5.2	0.6	-	5.3	9	-	3.8	0.1
Chrysene	0.7	122.1	0.3	1	4.3	0.7	5.2	1.1	-	2.7	10.8	6	6	33	19.8	-	8.1	1.2
Benzo[b]fluoranthene	0.6	46.2	-	0.9	-	0.4	1.7	-	-	1	10.5	2.9	3.5	52.9	25.3	-	8.6	3.3
Benzo[k]fluoranthene	-	11.8	-	-	-	-	-	-	-	-	4.2	0.3	-	196.5	9.5	-	1.9	-
Benzo[a]pyrene	-	18.8	-	-	-	-	0.6	-	-	0.6	7.1	0.6	-	17.9	19.1	-	3.8	-
Indeno[1,2,3-cd]pyrene	-	26.2	-	-	-	-	-	-	-	-	6.5	-	-	66.8	16.3	-	3.3	-
Dibenz[a,h]anthracene	-	8	-	-	-	-	-	-	-	-	-	-	-	-	2.3	-	-	-
Benzo[g,h,i]perylene	1.9	35.4	-	-	-	-	-	-	-	-	5.7	-	-	20.9	16.4	-	4.8	2.6
ΣPAH	3.2	339.6	5.4	41.9	133	31.5	46.6	28.6	23	31.7	199.1	54.7	95.1	666.7	176.9	43.9	162.1	116.3
BaP Equivalents	0.7	79.0	3.0E-03	1.3	0.6	0.7	2.4	0.3	0.2	1.5	19.7	3.5	3.8	99.8	50.7	0.3	13.8	4.3

Detected concentrations in samples that were below the minimum detection limit had a value substituted with half the detection limit. Values that were not detected are assumed 0 and marked with a -.

### Atmospheric PAH contamination

**Figure 3:**
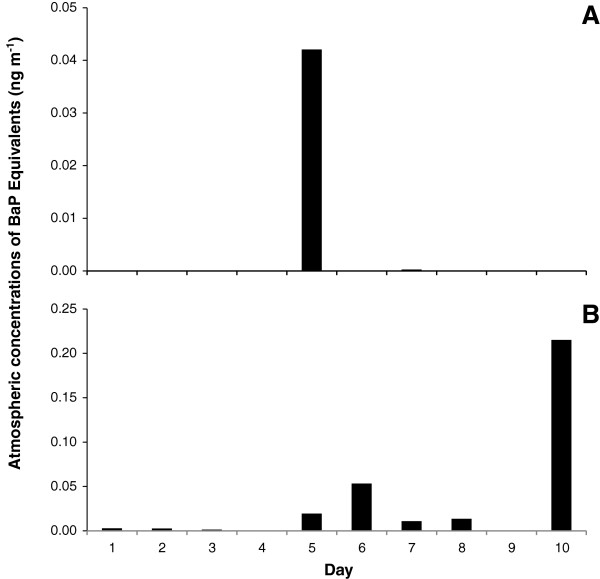
**Atmospheric concentrations of PAHs expressed as benzo[*a*]pyrene equivalents.** Panel **A** shows particulate PAH concentrations, and Panel **B** shows vapor-phase PAH concentrations.

High-volume air sampling collected particulate and vapour-phase PAH samples over ten 24 hour sampling periods (Figure 3). The mean concentrations of BaP equivalents were 0.0043 ± 0.013 ng m^-3^ and 0.032 ± 0.007 ng m^-3^ for the particulate and vapour phases, respectively. The vapour phase PAH concentration, expressed as BaP equivalents, is an order of magnitude greater than the particle-associated PAH levels. There was no correlation between particulate and vapour-phase PAH concentrations. The maximum particulate PAH concentration of 0.042 ng m^-3^ was obtained on the fifth day; the maximum gaseous PAH concentration of 0.22 ng m^-3^ was obtained on the tenth day.

### Cancer risk via soil ingestion

Our earlier work [22] indicated that the 95th percentile soil ingestion rate is 361 mg d^-1^, and the median rate is 37 mg d^-1^ the former determined using Si as the soil tracer and the latter determined using the mean of Al and Si soil tracers. Using a deterministic risk assessment approach, the calculated excess lifetime cancer risk at the maximum PAH concentration observed in this study was 3.29E-06 at the 95th percentile soil ingestion rate, assuming exposure for 365 days per year [28]. In a Canadian climate, daily exposure for a full year seems unlikely because of the relatively harsh winter. A second exposure frequency of 153 days a year was also employed for the risk assessment. This value represents daily exposure for the period from May through September, and the excess lifetime cancer risk associated with this exposure at 361 mg d^-1^ soil ingestion rate, and the maximum BaP equivalent soil concentration provided a value of 1.38E-06. Thus, there is no significant increase in the risk of cancer at this high soil ingestion rate; and moreover, the median soil ingestion rate the lifetime cancer risk was still substantially below 1E-05 (i.e., 1.41E-07). Furthermore, the mean BaP equivalent concentration of the collected Cold Lake soil samples (i.e., 15.7 ng g^-1^) also yielded risk values that are substantially below 1E-05 (i.e., 5.17E-07 at the 95th soil ingestion rate of 361 mg d^-1^ and 365 day yr^-1^ exposure).

**Figure 4:**
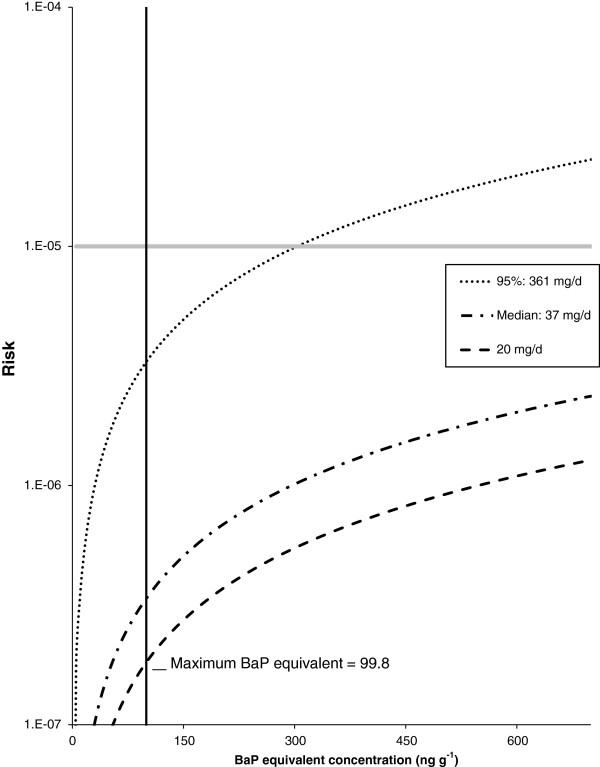
**Calculated excess life time cancer risk as a function of soil PAH concentration (expressed as BaP equivalents) for different soil ingestion rates.** Assumed exposure frequency of 365 days per year. The solid grey line denotes an excess lifetime risk of 1 extra cancer case per 100,000 people. This 1E-05 risk occurs at a BaP concentration of 304 ng g^-1^, 2962 ng g^-1^, and 5479 ng g^-1^ for soil ingestion rates of 361 mg d^-1^, 37 mg d^-1^, and 20 mg d^-1^, respectively. The solid black vertical line denotes the maximum BaP equivalent concentration. Two of the chosen soil ingestion rates are the 95th percentile (361 mg d^-1^) and median rates (37 mg d^-1^) from the Cold Lake soil ingestion study. The third value (i.e., 20 mg d^-1^) is the ingestion rate recommended by Health Canada for adult HHRA of contaminated sites.

**Figure 5:**
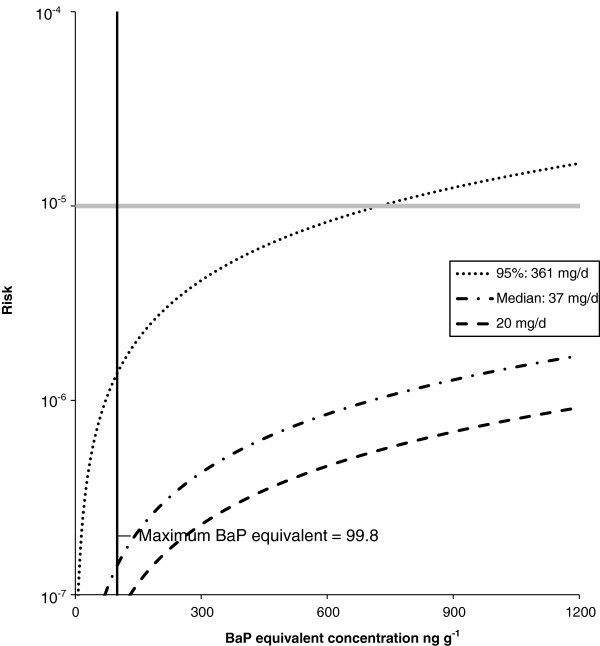
**Calculated excess life time cancer risk as a function of soil PAH concentration (expressed as BaP equivalents) for different soil ingestion rates.** Assumed exposure frequency of 153 days per year. The solid grey line denotes an excess lifetime risk of 1 extra cancer case per 100,000 people. This 1E-05 risk occurs at a BaP concentration of 724 ng g^-1^, 7066 ng g^-1^, and 13072 ng g^-1^ for soil ingestion rates of 361 mg d^-1^, 37 mg d^-1^, and 20 mg d^-1^, respectively. The solid black vertical line denotes the maximum BaP equivalent concentration. Two of the chosen soil ingestion rates are the 95th percentile (361 mg d^-1^) and median rates (37 mg d^-1^) from the Cold Lake soil ingestion study. The third value (i.e., 20 mg d^-1^) is the ingestion rate recommended by Health Canada for adult HHRA of contaminated sites.

Not surprisingly, the soil ingestion rate employed has a large effect on calculated excess lifetime cancer risk. At an assumed 365 days per year exposure (Figure 4) a significant increase in cancer risk occurs at a soil concentration of 304 ng g^-1^ BaP equivalents for the 95th percentile soil ingestion rate. If lifetime cancer risk is calculated using the median soil ingestion rate of 37 mg d^-1^, lifetime cancer risk would meet the 1 case per 100,000 threshold at a BaP equivalent soil concentration of 2962 ng g^-1^. The adult soil ingestion rate of 20 mg d^-1^ recommended by Health Canada for HHRA of contaminated sites, would require a soil concentration of 5479 ng g^-1^ of BaP equivalents to reach an increased cancer risk of 1 case per 100,000 people. Predictably, with an assumed exposure frequency of 153 days per year (Figure 5), which would represent non-winter months from May through September only, excess lifetime cancer risk in excess of 1E-05 would only be observed at much higher PAH concentrations. Using an EF of 153 day yr^-1^, at soil ingestion rates of 361 mg d^-1^, 37 mg d^-1^, and 20 mg d^-1^, the 1.0E-05 cancer risk threshold occurs at BaP equivalent concentrations of 724 ng g^-1^, 7066 ng g^-1^ and 13072 ng g^-1^ respectively.

The BaP oral slope factor used in this study provides estimates of the risk of gastric (stomach) cancer through ingestion. In Alberta, Canada annual age-standardized new cases of stomach cancer are 7 per 100,000 [36]. Therefore, even at the maximum PAH concentration in this study, the median soil ingestion rate of 37 mg d^-1^ does not significantly increase the risk of gastric cancer beyond the current background level (i.e., 7 cases per 100,000). Even at higher soil ingestion rates, there is no significant increase in excess cancer risk (Table 2). Using the 90th percentile soil ingestion rate of 152 mg d^-1^ and assumption of 365 d yr^-1^ exposure, the risk assessment predicts less than 0.1 new cancer cases per 100,000 over existing background. Using the 95th percentile soil ingestion rate of 361 mg d^-1^, the predicted excess risk is still less than 0.1 additional case per 100,000 people for an EF of 153 d yr^-1^. Thus, the results obtained, which are summarized in Table 2, indicate that the increased risk of gastric cancer associated with the median and mean PAH concentrations found in the soils from the Cold Lake region is low and may be considered negligible.

**Table 2:** Rates of gastric cancer (per 100,000 people) in Alberta, and the calculated increases in cancer incidence associated with exposure to contaminated soils in Cold Lake, Alberta

**Stomach cancer**	**New cases per 100,000**
Age-standardized incidence^a^	7
Age-standardized mortality^a^	4
Maximum PAH exposure 361 mg/d and 365 days/yr exposure frequency	7
Maximum PAH exposure 361 mg/d and 153 days/yr exposure frequency	7
Maximum PAH exposure 152 mg/d and 365 days/yr exposure frequency	7
Maximum PAH exposure 152 mg/d and 153 days/yr exposure frequency	7
Maximum PAH exposure 37 mg/d and 365 days/yr exposure frequency	7

a: [32].

Calculated cases per 100,000 are the sum of the current background rates and the additional cases associated with exposure to contaminated soil.

The excess cancer risk estimates shown above were compared to risk estimates based on published PAH levels for four other oil regions, using the Health Canada adult soil ingestion rate for HHRA of 20 mg d^-1^. The results for the Niger Delta, Nigeria; Texas, USA; Vojvodina, Serbia; and Mathura, India and are summarized in Table 3. Although these regions had much higher PAH concentrations than the Cold Lake region, there calculated excess cancer risk could still be termed negligible. The Texas, USA location had the greatest cancer risk level with 1.12E-05 for exposure at the maximum measured BaP equivalent concentration, and the only location above the Health Canada 1E-05 threshold. It is important to note that the US EPA typically uses a 1E-06 cancer risk threshold, and using this threshold, the calculated excess lifetime cancer risk values for Vojvodina, Serbia, and Niger Delta, Nigeria, as well as the Texas, USA location.

**Table 3:** Comparison of BaP equivalent concentrations and lifetime cancer risk estimates for different locations reported in the literature

		**BaP Equivalent**^**a **^**(mg kg**^**-1**^**)**		**Lifetime cancer risk**	
**Location**	**n**	**Maximum**	**Mean**	**Maximum**	**Mean**
Niger Delta, Nigeria^b^	8	5.3	2.0	4.05E-06	1.50E-06
Texas, USA^c^	3	14.7	11.0	1.12E-05	8.41E-06
Vojvodina, Serbia^d^	7	8.2	2.6	6.28E-06	1.98E-06
Mathura, India^e^	29	-	0.34	-	2.59E-07
Cold Lake, Canada	18	0.1	0.02	7.63E-08	1.20E-08

a: PAH concentrations converted to BaP equivalent concentrations using toxic equivalency factors [24].

b: [34].

c: [33].

d: [35].

e: [37].

Cancer risk was assessed using the maximum Cs, an ED of 70 years, an EF of 153 days, BW of 80 kg, an AT of 25550 days, and CSF_oral_ of 7.3 (mg kg^-1^ d^-1^)^-1^.

### Probabilistic risk assessment

**Figure 6:**
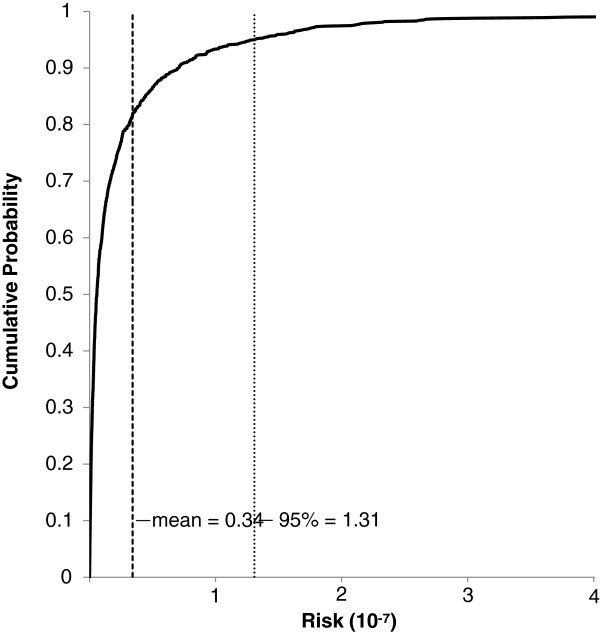
**Cumulative probability distribution of a Monte Carlo simulation based on 10,000 trials and the input variables from Table**4**.** The soil concentrations are the distribution of BaP equivalents measured at Cold Lake (Figure 1), the IR values were from Cold Lake soil ingestion study [22], and the exposure frequency is 153 days. The mean risk level is 3.42E-8, and the 9 % risk level is 1.31E-7.

**Figure 7:**
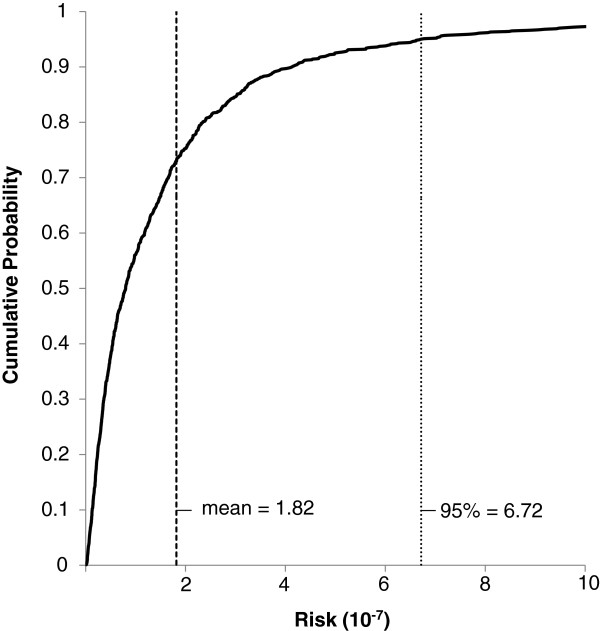
**Cumulative probability distribution of a Monte Carlo simulation based on 10,000 trials and the input variables from Table**5**.** The soil concentration was the highest BaP equivalent concentration measured at Cold Lake (Figure 1), IR values were from Cold Lake soil ingestion study [22], and the exposure frequency is 153 days. The mean risk level is 1.82E-7, and the 95% risk level is 6.72E-7.

Deterministic risk assessments (Figures 4 and 5) have been criticized for being too conservative (i.e., unnecessarily inflate risk). To account for the uncertainty of input variables, probabilistic risk assessments have been recommended [27,38]. Excess lifetime cancer risk was assessed using the probabilistic Monte Carlo simulation approach (Figure 6), the BaP equivalent soil concentrations, and the soil ingestion rates determined in the Cold Lake soil ingestion study [22]. The calculated lifetime cancer risk in the Cold Lake region were determined to be negligible with 95% of the population associated with a risk level almost tenfold below the 1E-05 threshold (input variables in Table 4 Excess lifetime cancer was also calculated using the maximum sampled BaP equivalent soil concentrations (Figure 7), and the aforementioned soil ingestion rates from the Cold Lake study (input variables Table 5). Again, calculated lifetime cancer risk is below the 1E-05 threshold, and thus negligible. A sensitivity analysis using Spearman’s rank correlation coefficient was carried out for the Monte Carlo risk analysis equation used for the probabilistic risk analysis [38]. The three variables examined were Cs, EF, and IR (Table 6). All three variables were positively correlated with lifetime cancer risk, with ingestion rate having the largest impact on lifetime cancer risk, closely followed by Cs, with EF having the smallest influence on risk.

**Table 4:** **Input variables for Monte Carlo simulation displayed in Figure**6

**Input variable**	**Distribution**	**Parameters**
Cs (mg/kg d.w. BaP equivalent)	Lognormal	Mean = 0.0157, SD = 0.0297
IR (mg/d)	Lognormal	Mean = 52, SD = 119
EF d/yr	Uniform	Min = max = 153
ED yr	Uniform	Min = max = 70
BW kg	Uniform	Min = max = 80
AT	Uniform	Min = max = 25550
CSF_oral_ (mg/kg-day)	Uniform	Min = max = 7.3

**Table 5:** **Input variables for Monte Carlo simulation displayed in Figure**7

**Input variable**	**Distribution**	**Parameters**
Cs (mg/kg d.w. BaP equivalent)	Uniform	Min = max = 0.0998
IR (mg/d)	Lognormal	Mean = 52 SD = 119
EF d/yr	Uniform	Min = max = 153
ED yr	Uniform	Min = max = 70
BW kg	Uniform	Min = max = 80
AT	Uniform	Min = max = 25550
CSF_oral_ (mg/kg-day)	Uniform	Min = max = 7.3

**Table 6:** Sensitivity analysis Spearman’s rank correlation coefficient values

**Input variable**	**Coefficient**
Cs mg kg^-1^	0.61
EF d yr^-1^	0.11
IR mg d^-1^	0.71

### Cancer risk via inhalation

Atmospheric samples of PAHs were collected near the base camp, approximately 10 km from the *in-situ* mining facilities, and inhalation rates recommended by Health Canada were used to assess excess lifetime cancer risk (Table 7) to recorded daily concentrations of BaP equivalents. The calculated excess lifetime cancer risk was far below the 1E-05 threshold, even at the maximum measured atmospheric BaP equivalent concentration of 2.15E-04 ng m^-3^ excess lifetime cancer risk through inhalation was 7.1E-10.

**Table 7:** Cancer risk estimates associated with inhalation of atmospheric PAHs in Cold Lake, Alberta

**BaP equivalent PAH concentration μg m**^**-3**^	**Lifetime cancer risk**
2.15E-04 (maximum)	7.10E-10
1.32E-08 (minimum)	4.37E-14
3.68E-05 (mean)	1.20E-10
7.08E-06 (median)	2.34E-11

10^-6^.

10^-7^.

## Discussion

### Soil and atmospheric PAH contamination

This study was designed to assess the excess lifetime cancer risk posed by exposures to PAHs in a region of Alberta that contains the world’s largest *in-situ* bitumen extraction operation, a process expected to overtake other conventional forms of petrochemical production. It was expected that PAH concentrations would follow a similar trend to that seen in Kelly et al. [6], where PAH concentrations were elevated up to 50 km from the oil sands upgraders in the Athabasca oil sands region. Although there is *in-situ* extraction in the Athabasca area, the region is dominated by bitumen extraction through open pit mining. Furthermore, the several upgrading and refining facilities in the Athabasca region are suspected of being major sources of atmospheric PAHs [6]. The Cold Lake region, despite being a large oil sands region, has a very different industrial landscape compared to the Athabasca region. The oil sands operations extract all bitumen using *in-situ* steam extraction methods, and the extracted bitumen is transported via pipeline to Lloydminster or Edmonton for upgrading and refining. The focus on *in-situ* extraction, and the lack of upgrading or oil refining in the Cold Lake region, may account for the comparatively low PAH concentrations observed in Cold Lake. The soil PAH concentrations recorded in this study suggest that *in-situ* oil sands development in the Cold Lake region do not make substantial contributions to local increases in soil PAH concentrations.

### Risk assessment

The results obtained failed to show an excess lifetime risk of cancer above the 1E-05 threshold, using the 95th percentile soil ingestion rate of 361 mg d^-1^[22] (Figure 4 and Table 2) and a 365 day per year EF. This EF will almost certainly contribute to an unrealistically inflated cancer risk due to the likely changes in seasonal soil ingestion rates. (i.e., an individual with an IR_soil_ at the 95th percentile would be expected to have a much lower IR_soil_ during winter months). Unfortunately, no published soil ingestion rate studies have investigated seasonal variations in ingestion rates. Thus, the likelihood, magnitude and direction of a seasonal change in IR_soil_ is unknown. It is reasonable to assume that soil ingestion rates during warmer months, when First Nations’ people who follow a traditional lifestyle spend greater amounts of time outside, would be substantially higher than during the winter months. Nonetheless, the estimated cancer risk associated with the reduced EF of 153 days per year is still below the 1E-05 threshold.

The Canadian Council of Ministers of the Environment (CCME) has set soil quality guidelines for BaP at 700 ng g^-1^ for the protection of human health [39,40]. The guidelines are based on an adult soil ingestion rate of 20 mg d^-1^, and this ingestion rate may not be applicable to all populations. Although the 37 mg d^-1^ median soil ingestion rate from the Cold Lake First Nations soil ingestion study is almost twice the recommended IR_soil_ for adults, the risk estimates presented here indicate that the excess lifetime cancer risk associated with levels below the CCME soil quality guidelines and the higher IR_soil_ are below 1E-05, and may be regarded as negligible. Probabilistic risk assessments give a better understanding of the cancer risk posed to a population, as opposed to simple point estimates based on measured Cold Lake soil ingestion rates (Figures 6 and 7). Nevertheless, probabilistic assessments also indicated that the excess lifetime cancer risk associated with exposures to soil PAH levels observed at Cold Lake are well below 1E-05, and can be regarded as negligible. The significance of this determination of negligible risk should not be overlooked since *in-situ* bitumen extraction is expected to overtake open pit mining as the dominant form of bitumen extraction in Alberta [15].

The calculated increases in cancer incidence that would be expected from inadvertent ingestion of contaminated soil at Cold Lake, even at the 95% IR_soil_ rate and maximum BaP equivalent soil concentrations (Table 2), are well below the background incidence of gastric cancer (i.e., 7E-05), and thus, the expected increase over background can also be considered to be negligible [36]. In contrast, a study that examined excess lifetime cancer risk from oral exposures to PAHs in settled house dust (i.e., inadvertent ingestion) noted far higher risk estimates. More specifically, for the maximum PAH concentration recorded in the study by Maertens et al. [41], and a 50 mg d^-1^ dust ingestion rate, the authors noted an increase of 27.4 extra gastric cancer cases per 100,000, compared to less than 1 excess per 100,000 people in this study for the maximum PAH concentration. Similarly, Williams et al. [42] investigated the excess cancer risk from exposure to settled house dust and soil at locations adjacent to parking lots sealed with coal-tar containing products. Using an IR distribution with a mean of 27 mg d^-1^, exposure to PAHs in settled house dust yielded an increase of 1.8 cases per 100,000 people, and 1.2 cases per 10,000 at the 50th and 95th percentiles, respectively. Exposure to soils adjacent to parking lots sealed with coal-tar containing products were associated with an even a greater increase of 7.3 cases per 100,000 at the 50th percentile, and 4.3 cases per 10,000 people at the 95th percentile. Although the studies by Maertens et al. [41] and Williams et al. [42] examined very different environments, they reinforce the notion that the calculated risks attributable to PAHs in soils near an *in-situ* oil sands facility might be reasonably regarded are indeed negligible.

It should be noted that other studies of excess lifetime cancer risk attributable to ingestion of PAHs in contaminated soil and street dust have also yielded values that can be considered negligible. For example, a study by Wang et al. [42] that calculated risk from exposures to PAHs in urban street dust revealed a mean risk from dust ingestion of 2.51E-6. The mean BaP equivalent concentration from the study by Wang et al. [42] was 0.47 mg kg^-1^, which is much greater than the average for Cold Lake soils of (i.e., 0.016 mg kg^-1^). Man et al. [43] used PAH levels in 12 different soils collected in Hong Kong to assess the excess lifetime cancer risk. At the median level, no soil yielded a cancer risk above the 1E-05 threshold, and only one site, near a car dismantling workshop yielded an excess cancer risk at the 95th percentile of 24 per 100,000. Among the other sites in the Man et al. [43] study that might be expected to be associated with cancer risk values greater than the 1E-05 threshold was an open burning site where large amounts of pyrogenic PAHs would be expected. Nevertheless, the calculated rates were below the 1E-05 threshold. Although only one location from the Man et al. study yielded cancer risk estimates above the 1E-05 threshold, PAH levels at seven of the sampled sites, which ranged from 0.090 mg kg^-1^ BaP equivalents to a maximum of 4.93 mg kg^-1^ near a car dismantling workshop, were above the Cold Lake average of 0.016 mg kg^-1^.

Despite the fact that the deterministic scenarios yielded risk values below the 1E-05 threshold, the deterministic risk assessments may still be expected to inflate risk values over the true lifetime cancer risk. In contrast, the probability distribution functions (PDF) (Figure 6) are likely to be far more representative of lifetime cancer risk in the Cold Lake region, since the PDF functions can account for the distribution of some or all the variables included in a risk assessment calculation [38]. For example, the soil ingestion rate likely varies across a population, as evidenced by the relatively high standard deviations noted in the companion soil ingestion study [22]. Additionally, PAH concentrations are also highly variable (Figure 2 and Table 1). The same risk assessment was run with both a deterministic and probabilistic approach. Although the deterministic assessment, which used an IR_soil_ of 37 mg d^-1^ and the mean soil PAH concentration, yielded a cancer risk of 2.22E-8, the probabilistic approach, which used a distribution for PAH soil concentration and IR_soil_ (Table 4), yielded a very similar mean cancer risk of 3.37E-8. Nevertheless, it is critical to note that the probabilistic assessment and the cumulative probability figure (i.e., Figure 6) provide an ability to determine that the mean risk level of 3.39E-8 accounts for more than 80% of the population. Thus, the expected probabilistic cancer risk level for most of the population is even lower than the level calculated using the deterministic equation [38]. In the absence of a PDF, the calculated risk may only represent a small part of the population.

Few published studies report soil PAH concentrations in petroleum extraction and refining regions; however, the concentrations that have been recorded are much greater than those found in the Cold Lake oil sands region (Table 3). More specifically, the mean BaP equivalent concentrations observed at other sites are at least an order of magnitude greater than those observed in this study, and only the mean concentration from a site in India, which reported a BaP equivalent concentration of 0.34 mg kg^-1^, was below the Canadian CCME guideline value [37]. The cancer risk estimates associated with the PAHs recorded at other petroleum extraction and refining locations are also much greater than those calculated for Cold Lake, but nevertheless, still below the Health Canada threshold. However, it should be emphasized that the industrial landscape at Cold Lake is different from the other examined locations. For example, with the exception of the Texas location, where soils were contaminated by faulty storage facilities, all the other locations with high PAH levels were in close proximity to refinery operations. This suggests that atmospheric release and deposition in the vicinity of oil refining operations contributes to regional soil contamination.

The sensitivity analysis (Table 6) shows that the soil ingestion rate metric has the largest effect on cancer risk estimation [27]. Sensitivity analysis data in other published studies are rare, but one study that also assessed cancer risk from PAHs through inhalation and dermal exposure, also found EF to have the lowest influence on lifetime cancer risk, relative to other variables [44]. Williams et al. [45] also conducted a sensitivity analysis on the Cs and IR (dust and soil) variables, and found that, for adults, the Cs parameter had the greatest influence on assessments that examined exposures to PAHs in soil or house dust adjacent to pavement where coal-tar containing products were not used. In contrast, if coal-tar containing sealant was used, then IR_soil_ had a larger influence on cancer risk estimation. These results are consistent with this study, where both IR and Cs were found to have similar influence on cancer risk estimation.

The cancer slope factor employed in this study is recommended by Health Canada. However, it is important to note that other jurisdictions and regulatory agencies may use different slope factors for determinations of excess lifetime cancer risk. For example, the California Office of Environmental Health Hazard Assessment (OEHHA) uses an oral slope factors of 2.9 (mg kg^-1^ d^-1^)^-1^, which is markedly different from the Health Canada value of 7.3 (mg kg^-1^ d^-1^). Nevertheless, with respect to the risk assessments conducted in this study, modest differences in slope factors would not significantly alter the excess lifetime cancer risk values associated with PAH exposures in Cold Lake (i.e., risk values still well below the 1E-05 threshold).

### Cancer risk via inhalation

The atmospheric concentrations of PAHs are also associated with risk estimates that are well below the threshold value of 1E-05. One published study reported rural atmospheric BaP equivalent concentrations in Canada as a median of 0.14 ng m^-3^, with major cities such as Toronto and Winnipeg having median BaP equivalent concentrations of 0.76 ng m^-3^ and 0.14 ng m^-3^, respectively, compared to the maximum BaP equivalent concentration measured in this study of 0.21 ng m^-3^[20]. In comparison, the World Health Organization recommends an atmospheric BaP concentrations of 0.12 ng m^-3^ to ensure that excess risk does not exceed 1 cancer case per 100,000 people [19,46], current guidelines from the Alberta Government require an annual average BaP atmospheric concentration of 0.30 ng m^-3^[47], and the Ontario Ministry of the Environment (MOE) specifies an Ambient Air Quality guideline of 1.1 ng m^-3^ over a 24 hour period [48]. Atmospheric BaP concentrations measured in the Cold Lake region are also typically lower than levels in major urban centers of Alberta. WBK & Associates Inc. [48] reported a 7 year median concentration of 0.12 ng m^-3^ for Calgary and 0.14 ng m^-3^ for Edmonton, with maximum annual average concentrations of 0.37 ng m^-3^ and 0.41 ng m^-3^ in Calgary and Edmonton, respectively. Only one of the observations recorded in the current is similar to typical urban BaP concentrations in Alberta, and the atmospheric concentration in Cold Lake can be regarded as fairly representative of a rural region in Alberta [18,49].

The low atmospheric PAH concentrations yielded lifetime cancer risk estimates from inhalation of atmospheric PAHs that are negligible for all concentrations, and even below the more conservative US EPA cancer risk threshold of 1E-6 (Table 7). It is difficult to know if the atmospheric PAH levels recorded over the 10 day sampling period are representative; nevertheless, the calculated risk estimates are negligible, and other studies that compared excess cancer risk from PAH exposures via inhalation and ingestion have noted that inhalation exposures make relatively small contributions to total risk [42,49].

## Conclusions

This study examined excess lifetime cancer risks posed by exposures to PAHs in soil and air at an area close to the world’s largest *in-situ* bitumen extraction area. The deterministic and probabilistic risk assessment, which employed IR_soil_ values from the companion First Nations soil ingestion study [22], both indicate that cancer risk is below the 1E-05 threshold, and can thus be considered negligible in most jurisdictions. Moreover, the PDF showed that 95% of the population is below the risk threshold of 1E-05. Additionally, the risk associated with PAH exposures via inhalation was also considered negligible. The hypothesis that there is an increased excess lifetime cancer risk from PAH exposures posed to First Nations people in the Cold Lake region is not supported by this study.

## Abbreviations

ASE: Accelerated solvent extraction; AT: Averaging time; BaP: Benzo[a]pyrene; BW: Body weight; CCME: Canadian Council of Ministers of the Environment; CF: Conversion factor; CS: Concentration; CSF: Cancer slope factor; ED: Exposure duration; EF: Exposure frequency; GFF: Glass fibre filter; HHRA: Human health risk assessment; IR: Intake rate; PAH: Polycyclic aromatic hydrocarbon; PEF: Potency equivalency factors; PDF: Probability density functions; PUF: Polyurethane foam cartridge; SD: Standard deviation.

## Competing interests

The authors have no conflict of interest to declare.

## Authors’ contributions

GI carried out the collection of soil and atmospheric samples, processed all samples, performed the data analyses, and prepared a draft of the manuscript. JMB conceived of the study, participated in its design, and helped draft the manuscript. JRD participated in study design, data analysis, and helped draft the manuscript. LEK participated in study design, method development and data analysis. PAW participated in study design, data analysis, and helped draft the manuscript. All authors have read and approved the final manuscript.

## Supplementary Material

Additional file 1: Table S1 PAH method detection limit and calculation for soil samples. Ce is analyte concentration, Ve, is injection analyte volume, Vs is average sample mass measured, R% is the average recovery rate of ^13^C labeled PAHs, and MDL is the calculated method detection limit. Click here for file

Additional file 2: Table S2 List of the 16 priority PAHs measured for this study and their Potency Equivalency Factors [24]. Click here for file

Additional file 3: Table S3 Method detection limit and calculation for particulate PAH samples measured on GFFs. Ce is analyte concentration, Ve, is injection analyte volume, Vs is average sample mass measured, R% is the average recovery rate of ^13^C labeled PAHs, and MDL is the calculated method detection limit. Click here for file

Additional file 4: Table S4 Method detection limit and calculation for gaseous PAH samples measured on PUF cartridges. Ce is analyte concentration, Ve, is injection analyte volume, Vs is average sample mass measured, R% is the average recovery rate of ^13^C labeled PAHs, and MDL is the calculated method detection limit. Click here for file
