# Supplementary material for: Cancer risk to First Nations’ people from exposure to polycyclic aromatic hydrocarbons near in-situ bitumen extraction in Cold Lake, Alberta
Source: Environ Health. 2014 Feb 12;13:7. doi: 10.1186/1476-069X-13-7 (PMC3930073; doi:10.1186/1476-069X-13-7)
Supplement: Additional file 1: Table S1 — PAH method detection limit and calculation for soil samples. Ce is analyte concentration, Ve, is injection analyte volume, Vs is average sample mass measured, R% is the average recovery rate of 13C labeled PAHs, and MDL is the calculated method detection limit. [file 1476-069X-13-7-S1.docx]

Table S1: List of the 16 priority PAHs measured for this study and their Potency Equivalency Factors ^(24)^.

| PAH Compound | Potency Equivalency Factor |
| --- | --- |
|  |  |
| Naphthalene | 0 |
|  |  |
| Acenaphthylene | 0.01 |
| Acenaphthene | 0 |
|  |  |
| Fluorene | 0 |
|  |  |
| Phenanthrene | 0 |
| Anthracene | 0.01 |
|  |  |
| Fluoranthene | 0.01 |
| Pyrene | 0 |
|  |  |
| Benz[a]anthracene | 0.1 |
|  |  |
| Chrysene | 0.01 |
|  |  |
| Benzo[b]fluoranthene | 1 |
|  |  |
| Benzo[k]fluoranthene | 0.1 |
|  |  |
| Benzo[a]pyrene | 1 |
|  |  |
| Indeno[1,2,3-cd]pyrene | 0.1 |
| Dibenz[a,h]anthracene | 1 |
|  |  |
| Benzo[g,h,i]perylene | 0.01 |
