# Supplementary material for: Cancer risk to First Nations’ people from exposure to polycyclic aromatic hydrocarbons near in-situ bitumen extraction in Cold Lake, Alberta
Source: Environ Health. 2014 Feb 12;13:7. doi: 10.1186/1476-069X-13-7 (PMC3930073; doi:10.1186/1476-069X-13-7)
Supplement: Additional file 2: Table S2 — List of the 16 priority PAHs measured for this study and their Potency Equivalency Factors [24]. [file 1476-069X-13-7-S2.docx]

Table S2: PAH method detection limit and calculation for soil samples. Ce is analyte concentration, Ve, is injection analyte volume, Vs is average sample mass measured, R% is the average recovery rate of ^13^C labeled PAHs, and MDL is the calculated method detection limit.

| PAH | Ce' (ng mL^-1^) | Ve (mL) | Vs (g) | R% | MDL (ng g^-1^) |
| --- | --- | --- | --- | --- | --- |
| Naphthalene | 0.343683 | 1 | 1.717341 | 24.42561 | 0.82 |
|  |  |  |  |  |  |
| Acenaphthylene | 0.076227 | 1 | 1.717341 | 24.33039 | 0.18 |
|  |  |  |  |  |  |
| Acenaphthene | 0.659067 | 1 | 1.717341 | 23.65808 | 1.62 |
| Fluorene | 0.21459 | 1 | 1.717341 | 40.199 | 0.31 |
| Phenanthrene | 0.155418 | 1 | 1.717341 | 33.65142 | 0.27 |
|  |  |  |  |  |  |
| Anthracene | 0.038777 | 1 | 1.717341 | 30.02193 | 0.08 |
|  |  |  |  |  |  |
| Fluoranthene | 0.099717 | 1 | 1.717341 | 45.36169 | 0.13 |
|  |  |  |  |  |  |
| Pyrene | 0.066034 | 1 | 1.717341 | 36.19563 | 0.11 |
|  |  |  |  |  |  |
| Benz[a]anthracene | 0.264983 | 1 | 1.717341 | 49.09347 | 0.31 |
|  |  |  |  |  |  |
| Chrysene | 0.152506 | 1 | 1.717341 | 57.78152 | 0.15 |
|  |  |  |  |  |  |
| Benzo[b]fluoranthene | 0.232417 | 1 | 1.717341 | 49.21396 | 0.27 |
| Benzo[k]fluoranthene | 0.266377 | 1 | 1.717341 | 61.1021 | 0.25 |
|  |  |  |  |  |  |
| Benzo[a]pyrene | 0.965446 | 1 | 1.717341 | 46.92114 | 1.20 |
| Ind[123cd[pyrene | 1.284471 | 1 | 1.717341 | 48.80843 | 1.53 |
|  |  |  |  |  |  |
| Dibenz(a,h)anthracene | 4.111251 | 1 | 1.717341 | 51.69556 | 4.63 |
|  |  |  |  |  |  |
| Benzo[ghi]perylene | 1.006194 | 1 | 1.717341 | 44.78616 | 1.31 |
