# Supplementary material for: Cancer risk to First Nations’ people from exposure to polycyclic aromatic hydrocarbons near in-situ bitumen extraction in Cold Lake, Alberta
Source: Environ Health. 2014 Feb 12;13:7. doi: 10.1186/1476-069X-13-7 (PMC3930073; doi:10.1186/1476-069X-13-7)
Supplement: Additional file 3: Table S3 — Method detection limit and calculation for particulate PAH samples measured on GFFs. Ce is analyte concentration, Ve, is injection analyte volume, Vs is average sample mass measured, R% is the average recovery rate of 13C labeled PAHs, and MDL is the calculated method detection limit. [file 1476-069X-13-7-S3.docx]

Table S3: Method detection limit and calculation for particulate PAH samples measured on GFFs. Ce is analyte concentration, Ve, is injection analyte volume, Vs is average sample mass measured, R% is the average recovery rate of ^13^C labeled PAHs, and MDL is the calculated method detection limit.

| PAH | Ce' (ng mL^-1^) | Ve (mL) | Vs (m^3^) | R% | MDL (pg m^-3^) |
| --- | --- | --- | --- | --- | --- |
|  |  |  |  |  |  |
| Naphthalene | 0.343682864 | 1 | 844.7095 | 28.04 | 1.45 |
|  |  |  |  |  |  |
| Acenaphthylene | 0.076226958 | 1 | 844.7095 | 38.25333 | 0.24 |
|  |  |  |  |  |  |
| Acenaphthene | 0.659066901 | 1 | 844.7095 | 52.68333 | 1.48 |
|  |  |  |  |  |  |
| Fluorene | 0.214590366 | 1 | 844.7095 | 52 | 0.49 |
|  |  |  |  |  |  |
| Phenanthrene | 0.155418091 | 1 | 844.7095 | 59.05167 | 0.31 |
|  |  |  |  |  |  |
| Anthracene | 0.038777257 | 1 | 844.7095 | 39.76833 | 0.12 |
|  |  |  |  |  |  |
| Fluoranthene | 0.099717419 | 1 | 844.7095 | 84.84 | 0.14 |
|  |  |  |  |  |  |
| Pyrene | 0.066033818 | 1 | 844.7095 | 63.11333 | 0.12 |
|  |  |  |  |  |  |
| Benz[a]anthracene | 0.264983112 | 1 | 844.7095 | 56.19 | 0.56 |
|  |  |  |  |  |  |
| Chrysene | 0.152505823 | 1 | 844.7095 | 72.34333 | 0.25 |
|  |  |  |  |  |  |
| Benzo[b]fluoranthene | 0.232417364 | 1 | 844.7095 | 75.84667 | 0.36 |
|  |  |  |  |  |  |
| Benzo[k]fluoranthene | 0.266376841 | 1 | 844.7095 | 75.49 | 0.42 |
|  |  |  |  |  |  |
| Benzo[a]pyrene | 0.965446267 | 1 | 844.7095 | 69.29 | 1.65 |
|  |  |  |  |  |  |
| Ind[123cd[pyrene | 1.284470887 | 1 | 844.7095 | 80.34 | 1.89 |
|  |  |  |  |  |  |
| Dibenz(a,h)anthracene | 4.111250603 | 1 | 844.7095 | 77.28333 | 6.30 |
|  |  |  |  |  |  |
| Benzo[ghi]perylene | 1.00619433 | 1 | 844.7095 | 84.56333 | 1.41 |
|  |  |  |  |  |  |
